# Burnout and coping mechanisms among healthcare professionals in central Uganda

**DOI:** 10.3389/fpsyt.2024.1373743

**Published:** 2024-04-15

**Authors:** Amir Kabunga, Eustes Kigongo, Ponsiano Okalo, Samson Udho, Anna Auma Grace, Raymond Tumwesigye, Anne Ruth Akello, Marvin Musinguzi, Walter Acup, Jannat Nabaziwa, Enos Mwirotsi Shikanga, Haliama Namata

**Affiliations:** ^1^ Department of Psychiatry, Faculty of Medicine, Lira University, Lira, Uganda; ^2^ Department of Environmental Health and Disease Control, Faculty of Public Health, Lira University, Lira, Uganda; ^3^ Department of Midwifery, Faculty of Nursing and Midwifery, Lira University, Lira, Uganda; ^4^ Department of Community Health, Faculty of Public Health, Lira University, Lira, Uganda; ^5^ Department of Education Psychology, Moi University, Eldoret, Kenya; ^6^ Department of Mental Health, Makerere University, Kampala, Uganda

**Keywords:** burnout, coping, healthcare workers, quality of life, Uganda

## Abstract

**Background:**

The escalating global prevalence of burnout among healthcare professionals poses a serious health concern. Recent studies focus on prevalence and predictors of burnout among healthcare providers, emphasizing the need for well-being interventions. This study investigates burnout and coping mechanisms among healthcare professionals in central Uganda, addressing the dearth of knowledge about coping strategies specific to the region.

**Methods:**

An analytical facility cross-sectional study was conducted in five healthcare facilities in central Uganda between June to July 2023. Participants included physicians, nurses, and technicians actively engaged in direct patient care. Data were collected using socio-demographic surveys, the Professional Quality of Life (ProQOL-5), and the Brief-COPE tools.

**Results:**

The study revealed a high prevalence of burnout, with 39.8% of participants experiencing significant levels. Active coping, positive reframing, and denial were negatively correlated with low burnout levels. Dysfunctional coping, specifically self-distraction and denial, showed positive correlations with average and high burnout levels. Emotion-focused coping mechanisms were not employed across burnout levels.

**Conclusions:**

The results emphasize the demanding nature of healthcare roles in the region and highlight the need for comprehensive, context-specific interventions to address burnout globally. While some healthcare professionals utilized adaptive strategies such as seeking social support, engaging in self-care activities, and utilizing problem-solving skills, others resorted to maladaptive coping mechanisms such as substance use and avoidance behaviors. This dichotomy highlights the need for targeted interventions to promote adaptive coping strategies and mitigate the negative impact of maladaptive behaviors on individual well-being and patient care.

## Introduction

The global prevalence of burnout among healthcare professionals has escalated, posing a serious health concern (1). Coined by Freudenberg in the 1970s, burnout manifests as emotional exhaustion, depersonalization, and a diminished sense of personal accomplishment, recognized as an occupational hazard in people-oriented professions (2). Extensive literature highlights risk factors, including heavy workloads, insufficient social support, role conflicts, and misalignment between personality and job requirements (3). Recent years have witnessed increased interest from mental health scholars, leading to numerous studies focusing on the prevalence and predictors of burnout among healthcare providers (4).

Burnout in the workplace has become a widespread health concern among healthcare professionals globally (1). Coined by Freudenberg in the 1970s, burnout is characterized by emotional exhaustion, depersonalization, and a diminished sense of personal accomplishment (2). It is acknowledged as an occupational hazard in people-oriented professions such as human services, education, and healthcare. Risk factors for burnout encompass a heavy workload, insufficient social support, role conflicts, communication or organizational issues, and a misalignment between personality and job requirements (3). In recent years, mental health scholars have shown an increasing interest in burnout-related issues, leading to numerous studies (5), particularly focusing on the prevalence and predictors of burnout among healthcare providers (4).

Studies demonstrate the significant impact of burnout on healthcare providers, with prevalence rates nearly equivalent among various roles (6). The repercussions extend to both professionals and patients, causing a decline in care quality, increased turnover, reduced clinical efforts, and substantial healthcare system costs (6). Physicians are almost twice as likely to contemplate leaving their positions, while a third of nurses cite burnout as a driving factor behind their decision to resign (7). Burnout not only affects the physical well-being of healthcare workers but is also associated with self-reported errors and elevated mortality rates among hospitalized patients (8). Consequently, there is a growing focus on healthcare workers’ well-being, urging the implementation of mitigation strategies to prevent burnout-associated risks (9).

This study is grounded in the Transactional Model of Stress and Coping proposed by Lazarus and Folkman, which offers a comprehensive framework for understanding how individuals perceive and react to stressors (10). This model is particularly relevant to our investigation into burnout and coping mechanisms among healthcare professionals, as it highlights that stress arises from an individual’s evaluation of a situation and their perceived ability to handle it. The model outlines two key processes: primary appraisal and secondary appraisal (11). Primary appraisal involves the initial assessment of whether a situation is insignificant, positive, or stressful. In our study of healthcare professionals in central Uganda, who contend with stressors like heavy workloads and limited resources, this primary appraisal is crucial. Secondary appraisal occurs when a situation is deemed stressful, prompting individuals to evaluate their coping resources and strategies (10). Our study aligns with this by examining coping mechanisms such as Problem-Focused Coping (active coping, planning, positive reframing) and Emotion-Focused Coping (use of emotional support, humor, acceptance), as well as Dysfunctional Coping (self-distraction, denial, substance use) as identified through the Brief-COPE tool (12).

The scenario of burnout is a significant and pressing issue for healthcare workers in central Uganda, impacting their well-being and the overall standard of treatment provided (13). The demanding nature of their responsibilities, coupled with limited resources and high patient loads, contributes to heightened stress levels and emotional exhaustion (14). Despite a well-documented history of burnout in the region (13), there is limited knowledge about the coping strategies employed by healthcare workers. Additionally, existing literature lacks the examination of the coping mechanisms specific to central Uganda, hindering the development of contextually relevant interventions. This underscores the need for a comprehensive study on coping mechanisms in central Uganda, essential for developing targeted interventions and support networks to mitigate burnout’s negative effects in this critical sector.

## Materials and methods

### Study design and participants

This was an analytical facility cross-sectional study conducted in five health facilities in central Uganda between June to July 2023. The participants comprised physicians, nurses, and technicians actively involved in direct patient care and holding crucial roles within the central Uganda health system. They were chosen from diverse medical facilities in the region, reflecting the varied tapestry of healthcare workers in that area. To be eligible for inclusion, participants had to be actively practicing physicians, nurses, or technicians engaged in direct patient care within the central Uganda health system. Exclusion criteria encompassed individuals on extended leave and participants with less than one year of experience, ensuring a comprehensive understanding of the potential impact of burnout on quality of life.

### Setting

The investigation was conducted in diverse healthcare environments, covering both public and private hospitals throughout central Uganda. Central Uganda, located in the heart of East Africa, showcases a mix of urban and rural landscapes and incorporates the capital city, Kampala. The area hosts a varied healthcare workforce, encompassing professionals from both public and private sectors, with government-owned facilities and private healthcare institutions playing crucial roles. Challenges encountered by the healthcare system include restricted resources, understaffing, and infrastructure limitations, which are indicative factors for burnout in the region (13).

### Sampling procedure and sample size

A sample size appropriate for cross-sectional study designs was employed to determine the requisite sample size. To ensure a maximum sample size, a 95% confidence interval (CI) and a 5% margin of error were utilized, with the estimated proportion (P) set at 50%. Furthermore, we increased the sample size to 550 healthcare workers, considering a 30% nonresponse rate and practical considerations. Employing a simple random sampling technique, participants were chosen from five prominent hospitals in central Uganda. Each of these hospitals received an equal allocation of the total sample, and proportionate sampling was employed for each job category within every hospital.

### Measurement instruments

This study employed three tools for data collection: the socio-demographic survey, the English version of the Professional Quality of Life (ProQOL-5), and the Brief-COPE (Coping, Orientation to Problem Experienced). Both the ProQOL-5 and Brief-COPE have been utilized in previous studies conducted in Uganda. Both PROQol and Brief-COPE have been used in previous studies in Uganda (12, 15).

The Brief-COPE consists of 14 sub-scales, each measured using two items on a 4-point Likert scale ranging from 0 to 3 (16). These sub-scales can be broadly categorized into approach/avoidant or adaptive/maladaptive forms of coping behaviors. The adaptive coping category encompasses 16 items, providing a potential score range of 0 to 48. It includes sub-scales such as active coping, planning, positive reframing, acceptance, humor, religion, using emotional support, and instrumental support. The maladaptive coping category comprises 12 items, with a possible score range of 0 to 36, and includes sub-scales like self-distraction, venting, substance use, denial, self-blame, behavioral, and disengagement. The overall reliability of the Brief-COPE tool in this study was found to be 0.79.

The ProQOL is a self-report questionnaire consisting of 30 items specifically crafted to evaluate burnout (10 items), compassion fatigue (10 items), and compassion satisfaction (10 items). A burnout score of 22 and below indicates a low level, 23–41 suggests an average level, and 42 or higher signifies a high level of burnout. In the present study, the Cronbach alpha value for the ProQOL is recorded as 0.89.

### Procedure

Following ethical approval, five research assistants, each possessing research experience, underwent training on tools for data collection, the study’s objectives, and ethical considerations. Subsequently, we reached out to officers from various health facilities in central Uganda, encompassing both public and private as well as rural and urban establishments, to apprise their healthcare staff of our study and seek their collaboration. Participants were briefed on the study’s objectives and urged to fill out a self-administered questionnaire. A trained psychologist was available to assist any participant in need. The survey, on average, took approximately 26 minutes to complete.

### Statistical analysis

The collected data underwent cleaning and post-entry coding in Microsoft Excel 2013. The cleaned dataset was then imported into STATA version 17 software for the formal analysis. Descriptive analysis was employed, summarizing normally distributed data as mean with standard deviation and skewed data as median with interquartile range. Categorical data was presented using simple frequencies and proportions. To evaluate the relationship between burnout and coping strategies, correlation analyses were conducted. For normally distributed data, the Pearson product moment correlation was employed, while Spearman rank correlation was used for skewed data. The outcomes were reported in terms of a correlation coefficient (r) and a p-value, with a significance level set at less than 0.05 to indicate a meaningful relationship between burnout levels and various coping mechanisms.

## Results

### Social demographic information

**Table 1** reveals that approximately half of the participants, specifically 279 individuals (50.9%), were below the age of 29. The majority of the participants, accounting for 340 (62.0%), were female, 352 individuals (64.2%) were living with a spouse or partner, and 242 individuals (44.2%) had worked for less than five years. In terms of occupation, the largest group consisted of nurses, totaling 255 individuals (46.5%), followed by technicians at 190 individuals (34.7%). More than half of the participants, specifically 318(58.0%), reported receiving support from management, and a significant portion, 343 (62.6%), did not experience workplace violence. The majority of the participants, 312 (56.9%), reported having adequate sleep at night.

**Table 1:** Demographic characteristics of healthcare workers in central Uganda (n=548).

Variable	Frequency (n)	Percentage (%)
Age		
<29 years	279	50.9
29-38 years	193	35.2
>39 years	76	13.9
Gender		
Female	340	62.0
Male	208	38.0
Marital status		
With spouse/partner	352	64.2
Without spouse/partner	196	35.8
Work experience		
<5 years	242	44.2
5-10 years	140	25.6
<10 years	166	30.3
Job category		
Physician	190	34.7
Nurse	255	46.5
Technician	103	18.8
Weekly work load		
30-38 hours	283	51.6
40 hours and above	265	48.4
Receive management support		
Yes	318	58.0
No	230	42.0
Experience workplace violence		
Yes	205	37.4
No	343	62.6
Have adequate sleep at night		
Yes	312	56.9
No	236	43.1

Levels of burnout among healthcare workers in central Uganda (n=548).

Burnout was measured as guided by the professional quality of life tool (17). **Table 2** shows that most of the participants 218(39.8%) had high level of burnout, and only 181(33.0%) had a low level of burnout.

**Table 2:** Levels of burnout among healthcare workers in central Uganda (n=548).

Burnout	Frequency (%)	95% Confidence Interval
Low	181 (33.0)	29.2-37.1
Average	149 (27.2)	23.6-31.1
High	218 (39.8)	35.8-44.0

### Coping strategies

Coping strategies were assessed using the Brief COPE tool (18), which consists of 28 items categorized into 14 subscales, each comprising two items. These subscales are further grouped into three main coping strategies: Emotion-Focused Coping (EFC), Problem-Focused Coping (PFC), and Dysfunctional Coping (DC) (19). In **Table 3**, the internal consistency of the tool was found to be satisfactory. The mean scores for Problem-Focused Coping, Emotion-Focused Coping, and Dysfunctional Coping were 2.61 ( ± 0.71), 2.43 ( ± 0.95), and 2.60 ( ± 0.42), respectively, with Problem-Focused Coping having the lowest mean score.

**Table 3:** Mean, standard deviation and reliability coefficients for coping strategies.

Coping strategy	Mean	Standard deviation	Alpha (α)
Problem-Focused coping	2.61	0.71	0.69
Active coping	2.77	0.73	0.88
Use of instrumental support	2.63	1.07	0.87
Positive reframing	2.73	0.99	0.87
Planning	2.32	1.09	0.85
Emotion-Focused coping	2.43	0.95	0.95
Use of emotional support	2.41	1.13	0.84
Venting	2.35	1.10	0.84
Humor	2.41	1.13	0.84
Acceptance	2.41	1.13	0.84
Self-blame	2.33	1.06	0.85
Religion	2.68	0.80	0.87
Dysfunctional coping	2.60	0.42	0.78
Self-distraction	2.80	0.76	0.90
Denial	2.51	0.60	0.86
Substance use	2.47	1.03	0.90
Behavioral disengagement	2.63	0.80	0.87

### Relationship between coping mechanisms and burnout

In **Table 4**, it is observed that low burnout levels showed negative correlations with active coping (*r*=-0.282, *p*=0.002), positive reframing (*r*=-0.295, *p*=0.001), self-distraction (*r*=-0.244, *p*=0.014), and denial (*r*=-0.244, *p*=0.014). Average burnout levels were positively correlated with dysfunctional coping, specifically self-distraction (*r*=0.347, *p*<0.001). High burnout levels were positively correlated with dysfunctional coping, particularly denial (*r*=0.349, *p*<0.001). Emotion-focused mechanisms were not employed at any level of burnout.

**Table 4:** Correlation between different coping mechanisms and levels of burnout.

Coping strategy	Low burnout		Average burnout		High burnout	
	r^#^	*P* value^#^	r^#^	*P* value^#^	r^+^	*P* value^+^
Problem-Focused coping	-0.346	<0.001*	0.176	0.477	0.013	0.850
Active coping	-0.282	0.002*	0.334	0.001	0.030	0.664
Use of instrumental support	-0.191	0.149	0.033	1.000	0.027	0.697
Positive reframing	-0.295	0.001*	0.112	1.000	-0.023	0.697
Planning	-0.213	0.061	0.014	1.000	0.015	0.829
Emotion-Focused coping	-0.197	0.217	0.039	1.000	0.035	0.611
Use of emotional support	-0.218	0.090	0.001	1.000	0.027	0.695
Venting	-0.208	0.136	-0.008	1.000	0.026	0.706
Humor	-0.218	0.090	0.001	1.000	0.024	0.721
Acceptance	-0.218	0.090	0.001	1.000	0.028	0.682
Self-blame	-0.123	1.000	0.018	1.000	0.042	0.538
Religion	-0.031	1.000	0.186	0.643	0.032	0.635
Dysfunctional coping	0.128	1.000	0.039	1.000	0.198	0.003*
Self-distraction	0.393	<0.001*	0.347	<0.001*	0.116	0.088
Denial	-0.244	0.014*	-0.181	0.408	0.349	<0.001*
Substance use	0.137	0.979	0.111	1.000	0.042	0.534
Behavioral disengagement	0.008	1.000	0.163	0.715	0.051	0.454

#: performed Spearman correlation, +: performed Pearson correlation, *significant p<0.05.

## Discussion

The study focused on examining burnout and coping mechanisms among healthcare professionals in central Uganda. The results indicated that 39.8% of healthcare workers experienced high levels of burnout. The findings revealed that active coping, positive reframing, and denial were negatively correlated with low burnout levels, while dysfunctional coping, specifically self-distraction and denial, showed positive correlations with average and high burnout levels.

Our findings indicate a concerning prevalence of high burnout levels, with 39.8% of participants reporting experiencing significant levels of burnout. These results underscore the substantial challenges faced by healthcare workers in the region, emphasizing the demanding nature of their roles and the potential impact on well-being. Numerous studies align with these findings, highlighting the widespread issue of burnout among healthcare professionals globally (20, 21). While burnout is recognized as a universal concern in the healthcare sector, variations in rates across regions and settings suggest the influence of factors such as organizational culture and leadership support (15, 22). Factors such as organizational culture, leadership support, and the availability of mental health resources can influence burnout levels (15, 22). Discrepancies in results may also be attributed to variations in the measurement tools used to assess burnout, highlighting the importance of standardized methodologies in cross-cultural studies. The results underscore the need for comprehensive and context-specific interventions to mitigate burnout among healthcare professionals globally. Also, addressing burnout among healthcare professionals should be prioritized to ensure the well-being of the workforce, which, in turn, can positively impact the quality of patient care.

Our findings indicate a connection between lower levels of burnout and the utilization of specific negative coping mechanisms, particularly self-distraction, as shown by observed negative correlations. Healthcare professionals resorting to self-distraction are more likely to experience reduced levels of burnout. While these coping strategies may provide temporary relief from stressors, they may not address the root causes of burnout, highlighting the complexity of the relationship between coping mechanisms and burnout. This underscores the need for comprehensive interventions addressing both individual coping skills and systemic factors contributing to burnout, consistent with existing studies supporting these findings (23). Further research should explore alternative coping strategies to establish a more comprehensive understanding of the dynamics influencing burnout among healthcare professionals. Notably, the absence of emotion-focused coping mechanisms at any level of burnout indicates a lack of adaptive strategies, emphasizing the prevalence of negative coping approaches in this population.

Our results also revealed a significant positive correlation between high burnout levels and dysfunctional coping, specifically denial. This suggests that as burnout increases, healthcare professionals are more likely to engage in denial as a coping mechanism. Conversely, inverse correlations observed at low levels of burnout indicate that healthcare professionals do not utilize self-distraction or denial as coping mechanisms, implying that while these coping strategies may offer temporary reprieve from stressors, they might not address the root causes of burnout. These findings align with the Transactional Model’s proposition that effective coping strategies can mitigate the impact of stressors (10). The concerning implications of these findings highlight that denial may hinder effective stress management and potentially exacerbate burnout. This aligns with existing research emphasizing the importance of proactive addressing of burnout and promoting healthier coping strategies in healthcare settings (24).

Our study findings indicate a negative relationship between active coping and positive reframing with burnout levels among healthcare workers. The observed negative correlations suggest that healthcare professionals who adopt proactive and positive strategies for managing stress are likely to experience lower levels of burnout. Our study findings support the theoretical underpinnings of this model. We observed that healthcare workers who engage in active coping and positive reframing tend to have lower levels of burnout. This implies that creating a work environment that promotes and supports active coping and positive reframing may function as a potential preventive measure against burnout among healthcare professionals in central Uganda. While these results are consistent with previous research highlighting the protective role of adaptive coping mechanisms (25), it is essential to acknowledge potential contextual and cultural variations.

A significant positive correlation was observed between high burnout levels and dysfunctional coping, particularly self-distraction. This finding suggests that healthcare professionals resorting to self-distraction are more likely to experience higher levels of burnout. This result shows the importance of addressing maladaptive coping strategies in efforts to mitigate burnout among healthcare professionals in the region. Interventions should focus on promoting healthier coping mechanisms to prevent and alleviate burnout. Existing literature support these findings, as studies in other settings have demonstrated similar associations between dysfunctional coping and elevated burnout levels among healthcare professionals (26). However, it would be valuable to explore dissenting views or alternative coping strategies to ensure a comprehensive understanding of the relationship between coping mechanisms and burnout in this specific context.

### Strengths and limitations of the study

The study holds contextual relevance as it delves into the specific context of central Uganda, shedding light on burnout and coping mechanisms among healthcare professionals in the region. the incorporation of standardized tools such as ProQOL-5 and Brief-COPE enhances the reliability and comparability of the study’s findings. The diversity of participants, drawn from various medical facilities, enriches the study’s generalizability by capturing the varied experiences of healthcare professionals in central Uganda. additionally, the inclusion of this diverse group of participants ensures that the findings can be broadly applicable and representative of the broader healthcare settings in the region.

Despite these strengths, the study faces certain limitations. The cross-sectional nature of the design restricts the ability to establish causation or observe changes over time. To address this, future research adopting a longitudinal approach could offer a more in-depth exploration of the dynamics between burnout and coping mechanisms. Additionally, reliance on self-reported data introduces the potential for social desirability bias. Furthermore, while the study’s findings contribute significantly to understanding burnout in central Uganda, the limited generalizability of the results beyond this region should be acknowledged. Lastly, potential confounders, such as individual differences in resilience and external support systems, remain unaccounted for and could influence the observed burnout levels and coping mechanisms. Longitudinal studies are warranted to explore the trajectory of burnout among healthcare professionals over time, considering factors like personality traits, resilience, and external support systems. Understanding how burnout develops and manifests longitudinally can inform the timing of interventions for maximum effectiveness.

## Conclusion

The results emphasize the demanding nature of healthcare roles in the region and highlight the need for comprehensive, context-specific interventions to address burnout globally. A notable discovery in our research was the range of coping mechanisms employed by healthcare professionals. While some utilized adaptive strategies such as seeking social support, engaging in self-care activities, and utilizing problem-solving skills, others resorted to maladaptive coping mechanisms such as substance use and avoidance behaviors. This dichotomy highlights the need for targeted interventions to promote adaptive coping strategies and mitigate the negative impact of maladaptive behaviors on individual well-being and patient care.

## Data availability statement

The original contributions presented in the study are included in the article/supplementary material. Further inquiries can be directed to the corresponding author.

## Ethics statement

The studies involving humans were approved by Lira University research ethics committee. The studies were conducted in accordance with the local legislation and institutional requirements. The participants provided their written informed consent to participate in this study.

## Author contributions

AK: Conceptualization, Formal analysis, Methodology, Writing – original draft, Writing – review & editing. EK: Conceptualization, Formal analysis, Funding acquisition, Methodology, Resources, Writing – original draft, Writing – review & editing. PO: Conceptualization, Data curation, Investigation, Project administration, Resources, Software, Supervision, Validation, Visualization, Writing – original draft, Writing – review & editing. SU: Formal analysis, Funding acquisition, Project administration, Resources, Validation, Visualization, Writing – original draft, Writing – review & editing. AG: Data curation, Funding acquisition, Investigation, Resources, Software, Supervision, Visualization, Writing – original draft, Writing – review & editing. RT: Data curation, Investigation, Methodology, Validation, Visualization, Writing – original draft, Writing – review & editing. AA: Funding acquisition, Resources, Visualization, Writing – original draft, Writing – review & editing. MM: Project administration, Resources, Writing – original draft, Writing – review & editing. WA: Conceptualization, Data curation, Investigation, Methodology, Software, Supervision, Writing – original draft, Writing – review & editing. JN: Funding acquisition, Project administration, Resources, Validation, Visualization, Writing – original draft, Writing – review & editing. ES: Formal analysis, Funding acquisition, Resources, Software, Supervision, Validation, Writing – original draft, Writing – review & editing. HN: Funding acquisition, Project administration, Resources, Visualization, Writing – original draft, Writing – review & editing.
